# Identification of IQGAP1 as a SLC26A4 (Pendrin)-Binding Protein in the Kidney

**DOI:** 10.3389/fmolb.2022.874186

**Published:** 2022-05-05

**Authors:** Jie Xu, Sharon Barone, Mujan Varasteh Kia, L. Shannon Holliday, Kamyar Zahedi, Manoocher Soleimani

**Affiliations:** ^1^ Research Services, VA Medical Center, Albuquerque, NM, United States; ^2^ Department of Medicine, University of Cincinnati, Cincinnati, OH, United States; ^3^ Department of Medicine, University of New Mexico, Albuquerque, NM, United States; ^4^ Department of Orthodontics, University of Florida, Gainesville, FL, United States

**Keywords:** kidney tubules, collecting duct, bicarbonate secretion, chloride absorption, intercalated cells

## Abstract

**Background:** Several members of the SLC26A family of transporters, including SLC26A3 (DRA), SLC26A5 (prestin), SLC26A6 (PAT-1; CFEX) and SLC26A9, form multi-protein complexes with a number of molecules (e.g., cytoskeletal proteins, anchoring or adaptor proteins, cystic fibrosis transmembrane conductance regulator, and protein kinases). These interactions provide regulatory signals for these molecules. However, the identity of proteins that interact with the Cl^−^/HCO_3_^−^ exchanger, SLC26A4 (pendrin), have yet to be determined. The purpose of this study is to identify the protein(s) that interact with pendrin.

**Methods:** A yeast two hybrid (Y2H) system was employed to screen a mouse kidney cDNA library using the C-terminal fragment of SLC26A4 as bait. Immunofluorescence microscopic examination of kidney sections, as well as co-immunoprecipitation assays, were performed using affinity purified antibodies and kidney protein extracts to confirm the co-localization and interaction of pendrin and the identified binding partners. Co-expression studies were carried out in cultured cells to examine the effect of binding partners on pendrin trafficking and activity.

**Results:** The Y2H studies identified IQ motif-containing GTPase-activating protein 1 (IQGAP1) as a protein that binds to SLC26A4’s C-terminus. Co-immunoprecipitation experiments using affinity purified anti-IQGAP1 antibodies followed by western blot analysis of kidney protein eluates using pendrin-specific antibodies confirmed the interaction of pendrin and IQGAP1. Immunofluorescence microscopy studies demonstrated that IQGAP1 co-localizes with pendrin on the apical membrane of B-intercalated cells, whereas it shows basolateral expression in A-intercalated cells in the cortical collecting duct (CCD). Functional and confocal studies in HEK-293 cells, as well as confocal studies in MDCK cells, demonstrated that the co-transfection of pendrin and IQGAP1 shows strong co-localization of the two molecules on the plasma membrane along with enhanced Cl^−^/HCO_3_^−^ exchanger activity.

**Conclusion:** IQGAP1 was identified as a protein that binds to the C-terminus of pendrin in B-intercalated cells. IQGAP1 co-localized with pendrin on the apical membrane of B-intercalated cells. Co-expression of IQGAP1 with pendrin resulted in strong co-localization of the two molecules and increased the activity of pendrin in the plasma membrane in cultured cells. We propose that pendrin’s interaction with IQGAP1 may play a critical role in the regulation of CCD function and physiology, and that disruption of this interaction could contribute to altered pendrin trafficking and/or activity in pathophysiologic states.

## 1 Introduction

Complex biological systems are composed of networks of interacting proteins, which are crucial for all levels of cellular function, including signaling, metabolism and communication. Several members of the Slc26 family of anion transporters form multi-protein complexes with the cytoskeleton, anchoring proteins, PDZ adaptor proteins and certain protein kinases (Lohi et al., 2003; Ko et al., 2004; Rossmann et al., 2005; Thomson et al., 2005; Bertrand et al., 2009; Lee et al., 2012; Kim et al., 2012; Zheng et al., 2010; Hillesheim et al., 2007; Dossena et al., 2011). The formations of these complexes impart regulatory signals on ion transport by members of the SLC26 transporter family. While several studies have identified binding partners for SLC26A3 (DRA), SLC26A5 (prestin), SLC26A6 (PAT-1; CFEX) and SLC26A9 (Lohi et al., 2003; Ko et al., 2004; Thomson et al., 2005; Rossmann et al., 2005; Hillesheim et al., 2007; Bertrand et al., 2009; Kim et al., 2012; Lee et al., 2012; Zheng et al., 2010; Dossena et al., 2011), little information is available about the proteins that interact with SLC26A4/pendrin.

SLC26A4 is a Cl^−^/HCO_3_^−^ exchanger located on the apical membrane of non-A intercalated cells and plays an important role in bicarbonate secretion and chloride absorption in the kidney CCD (Soleimani et al., 2001; Royaux et al., 2001; Wall et al., 2003; Vallet et al., 2006; Bonar and Casey, 2008; Sindić et al., 2007; Amlal et al., 2010; Soleimani, 2013; Alper and Sharma, 2013; Wall and Weinstein, 2013; Mohebbi et al., 2013). The purpose of the current studies was to identify the binding partners of SLC26A4 in the kidney. Toward this end, yeast two hybrid (Y2H) screening was utilized to identify the proteins that bind to the C-terminal end of pendrin. The C-terminus was chosen as bait for these studies because it contains the Sulfate Transporter and anti-Sigma factor antagonist (STAS) domain, as well as residues that are important in pendrin function (Dossena et al., 2011; Alper and Sharma, 2013; Soleimani, 2013). Results were further confirmed by immunoprecipitation experiments and functional and confocal image analysis in cultured cells, kidney sections, and proteins. We identify IQGAP1 as a binding partner of pendrin in the kidney. The significance of the results will be discussed.

## 2 Materials and Methods

### 2.1 Yeast Two Hybrid Screening

**FIGURE 1:**
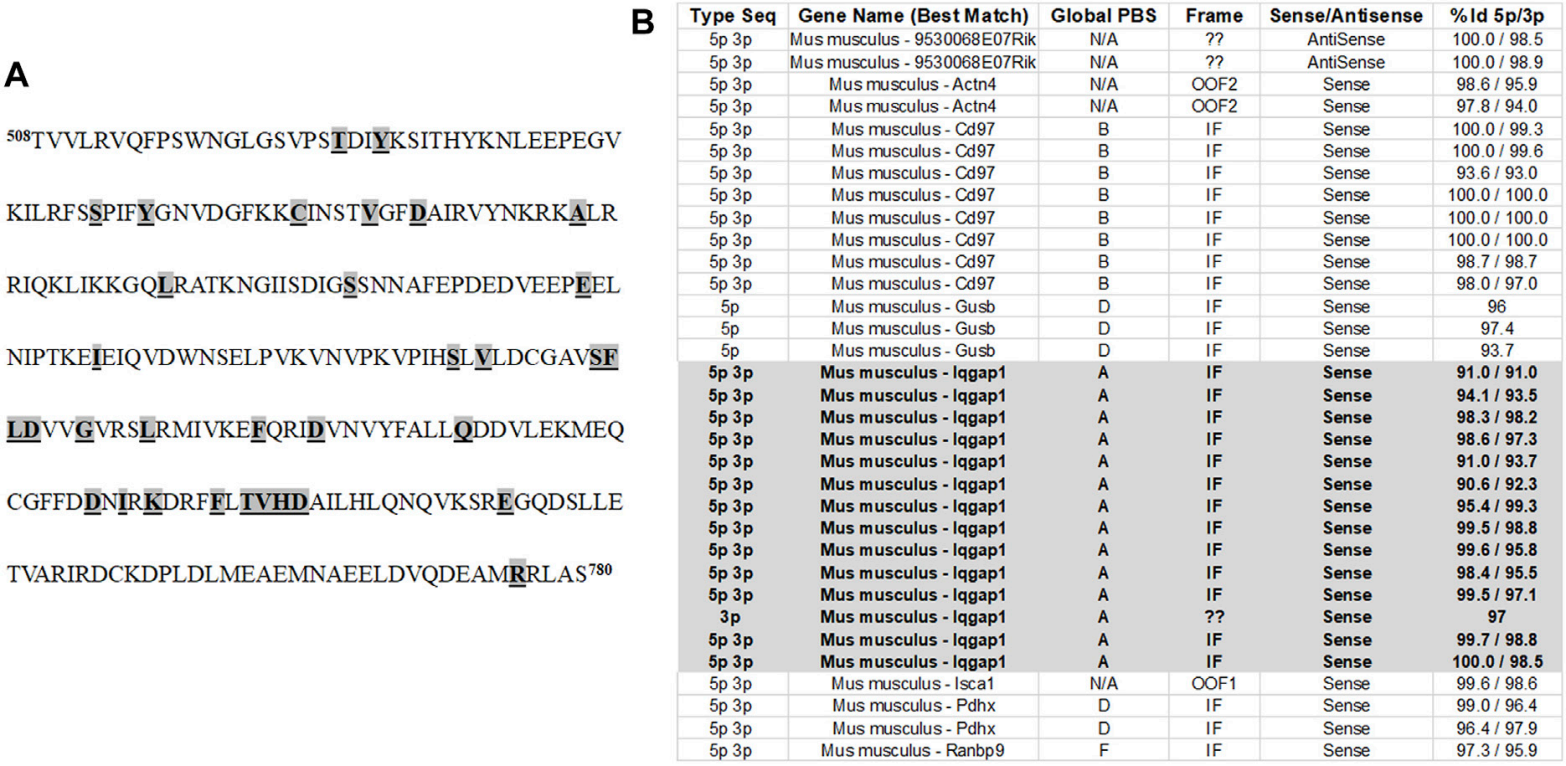
Identification of IQGAP1 as a pendrin binding partner. **(A)** The C-terminus of pendrin was used as bait in Y2H in order to identify the protein(s) that interact with pendrin. The C-terminus of mouse pendrin protein spanning amino acids 508–780 was used in Y2H studies in order to identify the proteins that interact with pendrin. This region was used because it contains the STAS domain. The missense mutations (highlighted, bolded and underlined) correspond to those of that affect the function of the human pendrin protein and are associated with Pendred Syndrome and DFNB4. **(B)** Our studies identified a total of 33 interactions between pendrin and prey sequences. Of these only 22 interactions were shown to be specific while others we excluded due to out of frame translation, reversed direction of the cloned sequence, or low affinity of the bait/prey interaction. The interaction with IQGAP1 represented 14 of the 22 significant interactions. Based on the Global PBS score, IQGAP/pendrin interaction had a very high degree of confidence. The Global PBS score is automatically computed through algorithms with the following scoring system: A) Very high confidence in the interaction; B) High confidence in the interaction; C) Good confidence in the interaction; D) Moderate confidence in the interaction; E) Interactions highly connected prey domains, warning of non-specific interaction; and F) Experimentally proven technical artifacts. In addition to IQGAP1, the Y2H system also identified CD97 as a protein with a “high confidence of interaction” giving it a Global PBS score of B. The relationship between pendrin and CD97 as a binding partner needs further examination. The % Id 5p/3p indicates the degree of identity to the putative binding protein.

In order to identify binding partners of mouse *Slc26a4* in the kidney, the Y2H screening (Joung, et al., 2000; Gietz et al., 1997) was employed. The intracellular portion of the c-terminal fragment of SLC26A4 (amino acids 508–780) was used as bait (Figure 1A). Briefly, the pBD-*Slc26a4* yeast two hybrid expression vector was constructed by PCR amplification of the cDNA fragment that codes for the SLC26A4 c-terminal fragment using the following primers: KUP2Hyb, 5′-GAC​TGT​GGT​CCT​GAG​AGT​TCA​G-3′, and KLOW2Hyb, 5′ TCA​GGA​AGC​AAG​TCT​ACG​CAT​G-3′. The sequence of the PCR amplified pendrin fragment was confirmed prior to initiation of the yeast two hybrid studies. The fragment was ligated into the *Sal*I restriction endonuclease site of pBD-GAL4 (Stratagene, La Jolla, CA, United States). A mouse kidney cDNA library (HybriZapTM two hybrid library, Stratagene) was co-transformed with pBD-*Slc26a4* into YRG-2-competent yeast cells. A total of 6 × 10^3^ interacting clones were identified by growth in selective media (Leu−, Trp−, His−), out of which 14 clones were determined to be positive when screened for β-gal expression. Plasmids from these clones were purified and co-transformed again with pBD-*Slc26a4* and with control plasmids in order to confirm the interaction. Cloned fragments were sequenced to confirm that they were in frame and without mutations.

### 2.2 Cloning of Full Length Human *SL26A4* and *IQGAP1*

For expression studies in HEK293 cells, the open reading frames of both *SLC26A4* (NM_000441) encompassing nucleotides 225–2,567 and *IQGAP1* (NM_003870) encompassing nucleotides 102–5,075 were amplified by PCR. Purified PCR products were sub-cloned into a pTarget ex-pression vector (Promega, Madison, WI, United States). Sequences and directionality of both sub-cloned fragments were confirmed.

### 2.3 Immunofluorescent Microscopic Analysis of Pendrin and IQGAP1 in the Kidney

The expression and localization of IQGAP1, SLC26A4 (pendrin), and H^+^-ATPase were characterized by immunofluorescence microscopic examination (Petrovic et al., 2006; Xu et al., 2011; Xu, et al., 2006) on paraffin-embedded mouse kidney sections using specific antibodies to SLC26A4, H^+^-ATPase B subunit, and IQGAP1.

### 2.4 Co-Immunoprecipitation and Western Analysis

For immunoprecipitation, 2 μg of mouse IQGAP1 antibody (SCBT, Dallas, TX, United States) was diluted in 200 μl of PBS with Tween-20 (PBS-T), and added to 50 μl of Dynabead Protein-G slurry (Invitrogen, Waltham, MA, United States). The mixture was subjected to rotation for 10 min at room temperature. The Dynabead-antibody complex was washed with PBS-T. Kidney lysates (200 µg in 100 μl of PBS-T) from wild-type (WT) and pendrin knockout (KO) (Amlal et al., 2010) were precleared by incubation with Dyna- bead Protein-G slurry for 30 min. The precleared lysates were added to the Dynabead-Ab complex. The mixture was subjected to rotation for 10 min at room temperature. The Dynabead-Ab-antigen complex was washed 3 times using 200 μl of PBS-T. The bound proteins were eluted by addition of 40 μl of 1:1 mixture of reducing laemmli and elution buffers and heating the samples for 10 min at 70 degrees C. The eluted proteins were subjected to western blot analysis using a mouse monoclonal anti-SLC26A4 antibody (LS Bio, Seattle, WA, United States).

### 2.5 Co-Expression of Pendrin and IQGAP1 in HEK293 and MDCK Cells

*SLC26A4* (pendrin) and *IQGAP1* expression vectors were used for transient transfection of HEK293 or MDCK cells. Briefly, cells were grown in 60 mm tissue culture plates or on coverslips. Monolayers (∼70% confluent) were transfected with 8 μg of the full-length SLC26A4 (pendrin), IQGAP1 or both expression vectors using Lipofectamine 2000, according to an established protocol (Li et al., 2004; Li et al., 2007). Cells were maintained at 37°C in a 5% CO_2_ atmosphere and were examined 48 h after transfection.

### 2.6 Confocal Microscopy

For confocal microscopy experiments, HEK293 or MDCK cells were grown on glass coverslips and transiently transfected with *Iqgap1*, *Slc26a4* (pendrin), or both expression vectors. The coverslips were fixed 48 h later with 4% paraformaldehyde 48 h after transfection. Fixed cells were labeled with IQGAP1 and SLC26A4 antibodies. The slides were observed using a Zeiss confocal 710. Z-stack images were obtained with LSM 5 Image software.

### 2.7 Intracellular pH Measurement

The intracellular pH (pH_i_) in HEK293 cells was determined by microfluorometry using the pH-sensitive fluoroprobe BCECF (Xu, et al., 2011; Petrovic, et al., 2003; Rahmati, et al., 2013). The cells were first perfused with a Cl^−^- and HCO_3_^−^-containing solution of the following composition (in mM): 115 NaCl, 25 Na-HCO_3_, 3 KCl, 1.8 CaCl_2_, 1 MgCl_2_, and 5 HEPES, pH 7.4, gassed with 5% CO_2_-95% O_2_. Once the baseline pH_i_ was established, the perfusate was then switched to a Cl^−^-free medium of the following composition (in mM): 115 Na^+^-gluconate, 25 NaHCO_3_, 3 KCl, 1.8 Ca^2+^-gluconate, 1 Mg^2+^-gluconate, and 5 HEPES, pH 7.4, and gassed with 5% CO_2_-95% O_2_. Upon pH_i_ stabilization in Cl^−^-free medium, cells were returned to the Cl^−^-containing solution. Values of pH_i_ were calculated from the fluorescence ratio (F480/F430) measured at 530 nm. The system was calibrated by the high-K^+^/nigericin technique.

### 2.8 Antibodies and Other Reagents

Polyclonal pendrin and H^+^-ATPase B1 subunit antibodies were generated in our laboratory as described (Petrovic, et al., 2006; Xu, et al., 2011; Xu, et al., 2006). Monoclonal H^+^-ATPase E subunit was a generous gift from Dr. Shannon Holliday. Monoclonal pendrin antibody was from LS Bio (Seattle, WA, United States). IQGAP1 antibody was purchased from Santa Cruz Biotechnology (Dallas, TX, United States). HRP-labeled goat anti-rabbit Ig was from PharMingen (San Diego, CA, United States). Dynabead protein-G immunoprecipitation kit was purchased from Thermo Fisher Scientific (Waltham, MA, United States). Western blot densitometry measurements were performed using Image-J software (National Institutes of Health, United States).

### 2.9 Statistical Analysis

The results for cell pH experiments are presented as means ± SE. Statistical significance between two experimental groups was determined by unpaired Student’s t-test. The statistical significance of results comparing multiple experimental groups was determined by ANOVA. A *p* < 0.05 was considered to be statistically significant.

## 3 Results

### 3.1 Identification of SLC26A4 Binding Partners

Figure 1A shows the amino acid sequence of the C-terminus of pendrin. This fragment encompasses the Sulfate Transporter and anti-Sigma factor antagonist (STAS) domain (amino acids 515–734), the intervening sequence (amino acids 574–652), and includes a number of disease-associated mutations (Bonar and Casey, 2008; Sindić et al., 2007; Soleimani, 2013; Alper and Sharma, 2013; Dossena, et al., 2011). The pendrin mutations included in Figure 1A are responsible for a variety of sensorineural hearing loss, including those found in patients with Pendred syndrome, as well as patients afflicted with non-syndromic hearing loss caused by enlarged vestibular aqueducts (EVA) (Dossena, et al., 2011; Roesch, et al., 2021). Y2H studies were used to identify the binding partners that interacted with the C-terminus of SLC26A4 (amino acids 508–780). These studies identified a total of 33 interactions, of these only 22 interactions were shown to be specific while others we excluded due to out of frame translation, reversed direction of the cloned sequence, or low affinity of the bait/prey interaction. The interaction with IQGAP1 represented 14 of the 22 significant interactions (Figure 1B). Our studies identified IQGAP1 as a protein that binds to the intracellular c-terminal portion of pendrin with a very strong degree of confidence (Figure 1B). There were two other proteins (Cluster of differentiation 97; Cd97 and beta-glucuronidase; Gusb) that were identified; however, their binding affinity was significantly less than IQGAP1.

IQGAP1 is a scaffolding protein with five identified protein binding domains (White, et al., 2012; Nammalwar, et al., 2015; Nauert, et al., 2003; Jacquemet, et al., 2013; Johnson, et al., 2013; Hedman, et al., 2015). It binds and/or stabilizes ezrin, CDC42 and RAC1, and interacts with a number of cytoskeletal and cell adhesion molecules (e.g., mDia and Cadherin) (White, et al., 2012; Nammalwar, et al., 2015; Nauert, et al., 2003; Jacquemet, et al., 2013; Johnson, et al., 2013; Hedman, et al., 2015). As such, IQGAP1 plays a role in the regulation of signal transduction, cytoskeleton, cell adhesion and cell cycle (White, et al., 2012; Hedman, et al., 2015).

### 3.2 Co-Localization of SLC26A4 (Pendrin) and IQGAP1 in the Kidney

**FIGURE 2:**
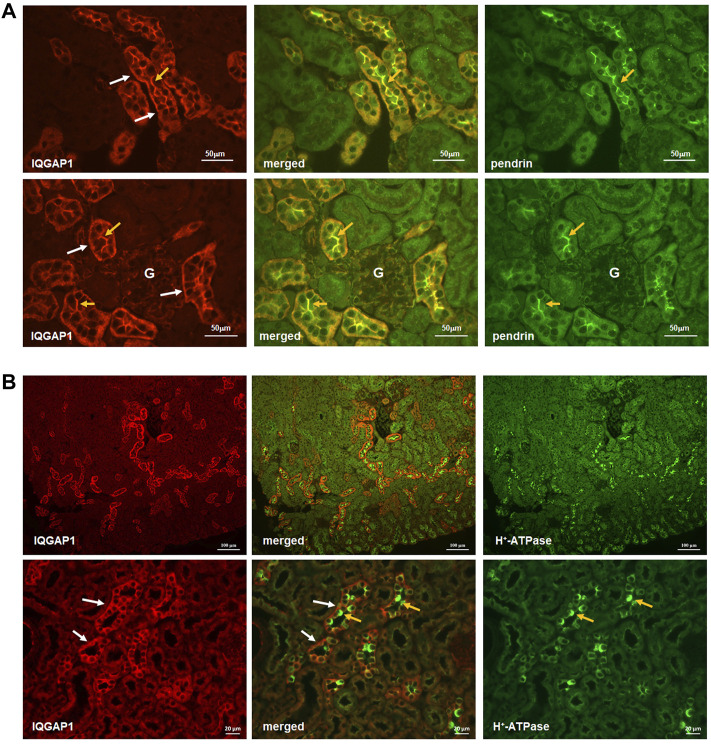
Co-localization IQGAP1, pendrin, and H^+^-ATPase in the kidney by immunofluorescence microscopy. **(A)** Top and bottom panels are immunofluorescence double labeling of mouse kidney sections with anti-IQGAP1 and anti-pendrin antibodies (×40 magnification). The expression of IQGAP1 is detected on the basolateral membrane in the of majority of cells in the CCD (Left Panels, top and bottom rows; white arrows). In addition, IQGAP1 shows apical localization in a subset of cells in CCD (Left Panels, top and bottom rows; orange arrows). The expression of pendrin is shown in right panels (orange arrows). Merged images (middle panels) demonstrates a remarkable co-localization of IQGAP1 and pendrin on the apical membrane of pendrin-expressing cells (orange arrows). “G” signifies glomerulus. **(B)** To determine the identity of tubular cells expressing IQGAP1, double immunofluorescence labeling with IQGAP1 and H^+^-ATPase antibodies was performed. As shown, IQGAP1 was detected in several cortical collecting duct and connecting tubules based on the presence of H^+^-ATPase, tubular morphology, and when the merged images were acquired (middle images in both top and bottom panels). There was occasional and faint expression of IQGAP1 on the basolateral mem-brane of the proximal tubule cells. In cortical collecting duct (CCD), IQGAP1 shows predominant localization on the basolateral membrane of most cells (bottom panels; white arrows). However, IQGAP1 also shows distinct localization on the apical membrane of a subset of intercalated cells (bottom panels; yellow arrows).

Given the results of Y2H system identifying IQGAP1 as a SLC26A4 binding protein, we sought to examine the localization of IQGAP1 vis-à-vis SLC26A4. Toward this end, double immunolocalization studies with SLC26A4 and IQGAP1 antibodies were performed in the kidney. IQGAP1 shows a predominant basolateral localization in various CCD cells (Figure 2A, left panels, top and bottom rows, white arrows). Certain cells also express IQGAP1 on their apical membrane (left panels, top and bottom rows, orange arrows). Merged images demonstrate a remarkable co-localization of SLC26A4 (pendrin) and IQGAP1 on the apical membrane of pendrin-expressing cells in the CCD (Figure 2A, merged images in the middle panel; top and bottom rows, orange arrows), consistent with the apical localization of IQGAP1 in B-intercalated cells.

Additional images depicting the localization of H^+^-ATPase vis-à-vis IQGAP1 are shown in the Figure 2B. In addition to confirming the predominant localization of IQGAP1 on the basolateral membrane of majority of CCD cells (Figure 2B; left bottom and middle panels, white arrows), these images clearly indicate the absence of IQGAP1 localization on the apical membrane of A-intercalated cells (Figure 2B; middle bottom panel, orange arrows). The images also show the apical localization of IQGAP1 in a subset of cells distinct from A-intercalated cells. Taken together with images in Figure 2, these studies indicate the apical localization of IQGAP1 in B-, but not A-, intercalated cells.

### 3.3 Co-Immunoprecipitation Studies

**FIGURE 3:**
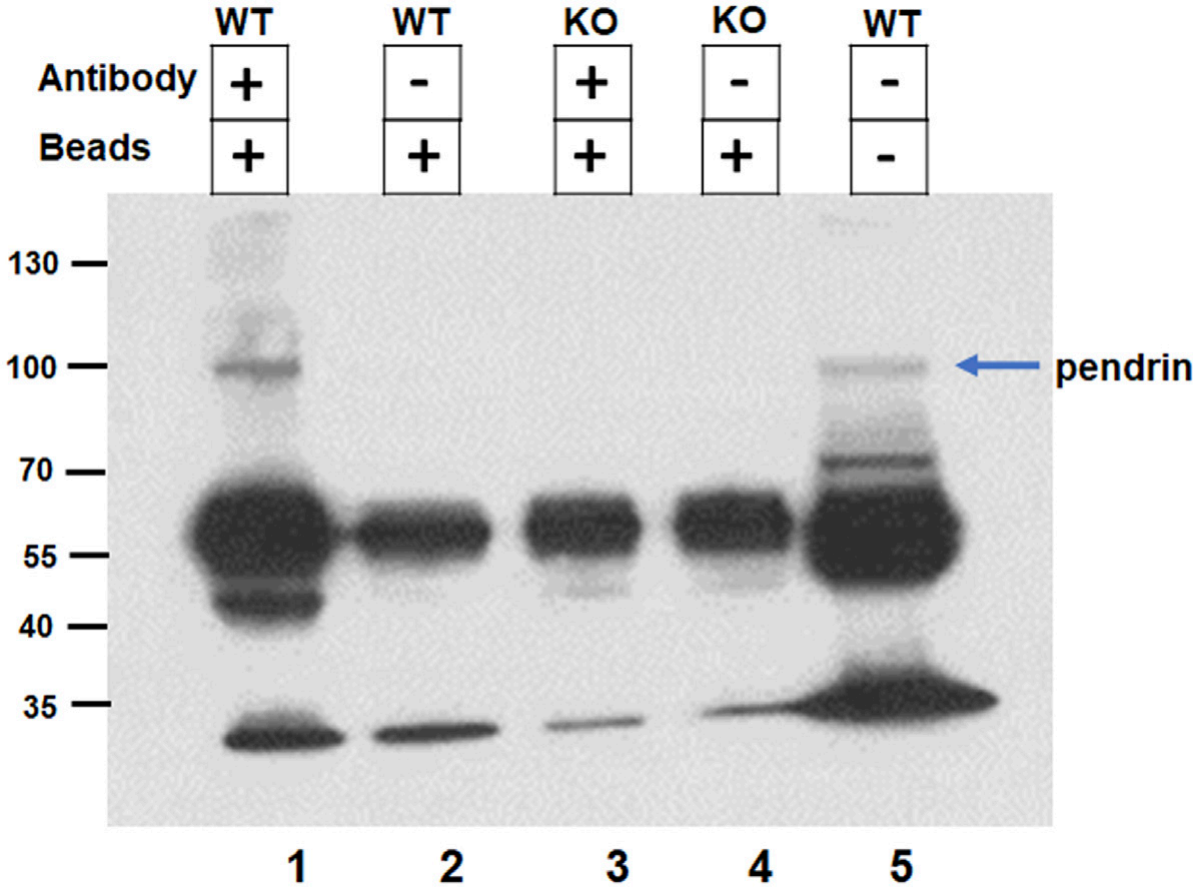
Confirmation of IQGAP1 and pendrin interaction by co-immunoprecipitation. The interaction of pendrin with IQGAP1 was confirmed by co-immunoprecipitation. Kidney extracts from wildtype and pendrin knockout animals were incubated with IQGAP1 antibody coated beads, the bound proteins were eluted, size fractionated and subjected to western blot analysis using anti-pendrin antibody. Lane 1) Binding of WT kidney extract with IQGAP1 antibody coated beads; Lane 2) Binding of WT kidney extract with G-protein coated beads; Lane 3) Binding of pendrin KO kidney extract with IQGAP1 coated beads; Lane 4) Binding of pendrin KO kidney extract with G-protein coated beads; and Lane 5) WT whole kidney extract. Blue arrow designates the 110 kDa band recognized by anti-pendrin antibody.

The interaction of SLC26A4 (pendrin) with IQGAP1 was further confirmed by co-immunoprecipitation. Kidney extracts from wildtype and pendrin knockout animals were incubated with IQGAP1 antibody coated beads, the bound proteins were eluted, size fractionated and subjected to western blot analysis using anti-pendrin antibodies. Our western blot results (Figure 3) show the presence of a band that corresponds in size (MW∼110 kDa) and reacts with anti-pendrin antibody in the whole kidney extract of WT mice (Lane 5), as well as kidney extracts of WT mice subjected to co-immunoprecipitation with anti-IQGAP1 antibody (Lane 1). This band was absent in the kidney extracts of pendrin KO mice that were subjected to co-immunoprecipitation using anti-IQGAP antibody (Lane 3). Non-specific binding of extract proteins to the matrix in the absence of anti-IQGAP antibody was minimal (Lanes 2 and 4).

### 3.4 Effect of IQGAP1 on Pendrin Activity and Localization in Cultured Cells

#### 3.4.1 Intracellular pH Studies

**FIGURE 4:**
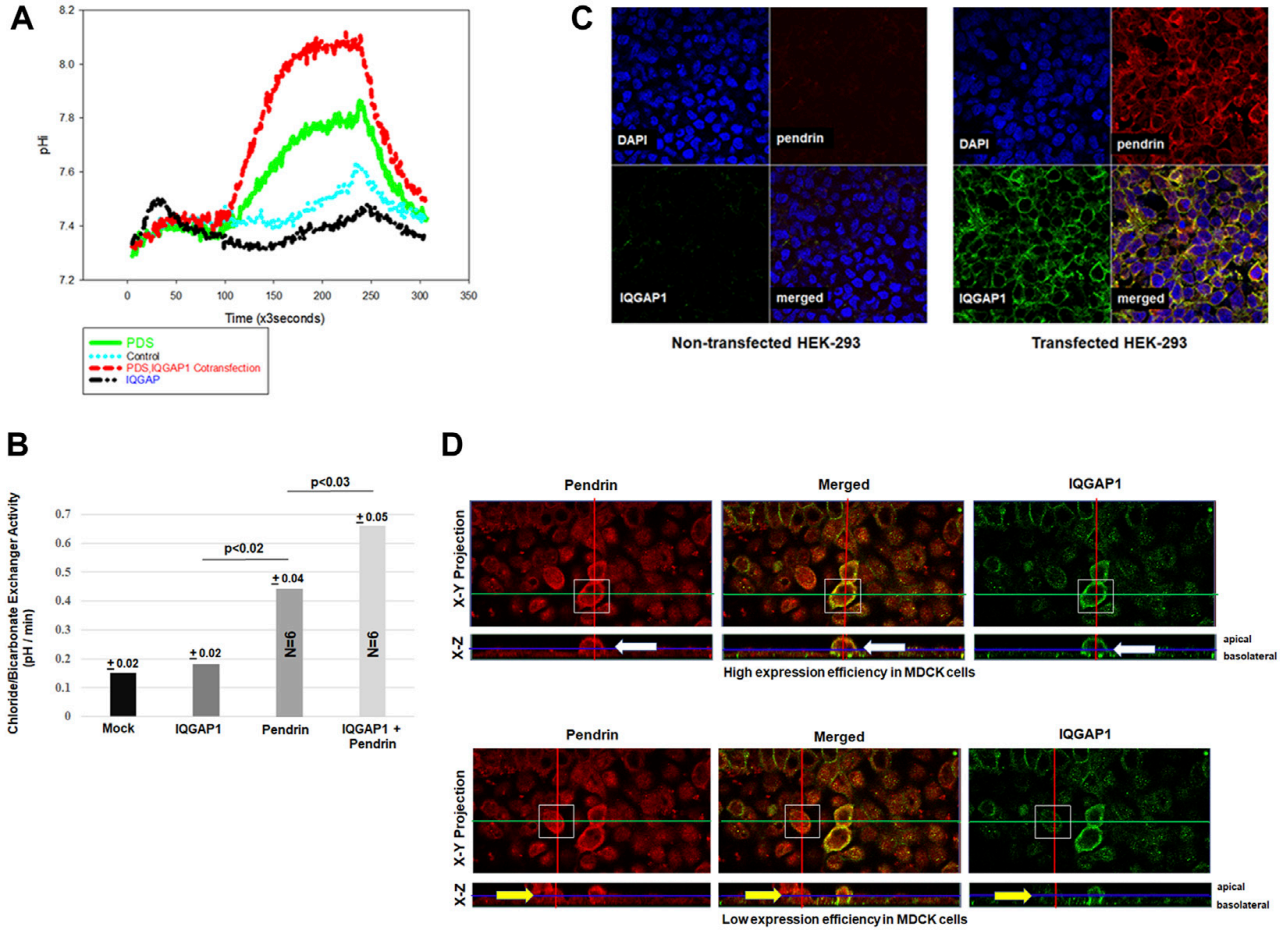
Effect of IQGAP1 on pendrin activity and expression in cultured cells. **(A)** Representative tracing demonstrating the Cl^−^/HCO_3_^−^ exchanger activity in transfected HEK293 cells. The mock transfected HEK293 cells were not significantly different vs. IQGAP1 transfected cells. The pH_i_ tracings in HEK293 cells transfected with pendrin alone show significant Cl^−^/HCO_3_^−^ exchanger activity compared to mock or IQGAP1 only transfected cells. HEK293 cells co-transfected with pendrin and IQGAP1 show significant enhancement in Cl^−^/HCO_3_^−^ exchanger activity vs. pendrin-transfected cells. **(B)** The summary of six separate experiments show significant enhancement in pendrin-mediated Cl^−^/HCO_3_^−^ exchanger activity in cells co-transfected with pendrin and IQGAP1 vs. pendrin alone transfected cells. IQGAP1 transfection in cells did not elicit any Cl^−^/HCO_3_^−^ exchanger activity when compared to mock transfected. **(C)** Representative confocal images of HEK293 cells transfected with IQGAP1, pendrin or IQGAP1 plus pendrin construct. Non-transfected cells (**C**; left top and bottom panels of non-transfected groups) did not show any expression of either pendrin or IQGAP1. HEK293 cells co-transfected with SLC26A4 and IQGAP1 (**C**; right top and bottom panels of transfected groups) show sharp co-localization of the two molecules on the plasma membrane of transfected cells (**C**; merged panel of transfected groups). **(D)** Representative confocal images of pendrin localization in high (**D**; upper panels) and low (**D**; bottom panels) IQGAP1-expressing MDCK cells. Both X-Y and X-Z projections are provided. White boxes highlight the cells that were analyzed. Cells with an elevated expression of IQGAP1 (**D**; top right and left panels, white arrow) have increased pendrin localization to the cell membrane. Whereas, low expression of IQGAP1 is associated with reduced membrane and increased intracellular localization of pendrin (**D**; bottom right and left panels, yellow arrow).

To determine if the interaction of IQGAP1 and SLC26A4 affects the activity of the latter, cultured HEK293 cells were co-transfected with *IQGAP1* and *SLC26A4* expression vectors and assayed for SLC26A4-mediated Cl^−^/HCO_3_^−^ exchanger activity. For comparison, cells transfected with pendrin or IQGAP1 expression vector alone were examined. The results in Figure 4 depict a representative intracellular pH (pH_i_) tracings (Figure 4A) and summary of multiple experiments (Figure 4B). These results indicate that the Cl^−^/HCO_3_^−^ exchanger activity in HEK293 cells transfected with the *IQGAP1* vector were not different than the mock transfected cells (background activity). HEK293 cells transfected with *SLC26A4* cDNA alone displayed significant Cl^−^/HCO_3_^−^ exchanger activity (Figures 4A,B). However, when HEK293 cells were co-transfected with both *SLC26A4* and *IQGAP1* expression constructs there was a significant enhancement in their Cl^−^/HCO_3_^−^ exchanger activity when compared to pendrin-transfected cells alone (Figures 4A,B).

#### 3.4.2 Pendrin Expression in Cultured Cells

Confocal microscopy was performed to examine the impact of IQGAP1 expression on SLC26A4 (pendrin) distribution in cultured cells. As indicated, the non-transfected cells (Figure 4C; left top and bottom panels of non-transfected groups) did not show any expression of either pendrin or IQGAP1 as verified by confocal microscopy. The results further indicate that HEK293 cells co-transfected with pendrin and IQGAP1 (Figure 4C; left top and bottom panels of transfected groups) show sharp co-localization of IQGAP1 and pendrin in the merged image of the transfected panel (Figure 4C; merged panel of transfected groups). Additional experiments were conducted to verify the interaction between pendrin and IQGAP1 in a polarized cell model and the role of this interaction in the localization of pendrin. Towards this end, confocal images of MDCK cells transiently transfected with both pendrin and IQGAP1 expression vectors are shown in Figure 4D. One limitation with expression studies in MDCK cells is a low or variable transfection efficiency. Therefore, we examined cells showing either high or low IQGAP1 expression. Our results indicate that cells with high expression levels of IQGAP1 (Figure 4D; top right panel, white arrow) have increased pendrin membrane localization (Figure 4D; top left panel, white arrow). As indicated, cells showing a strong abundance of IQGAP1 demonstrate discrete apical co-localization of IQGAP1 and pendrin. In contrast, cells expressing low levels of IQGAP1 show reduced membrane and increased intracellular localization of pendrin (Figure 4D; bottom right and left panels, yellow arrow).

## 4 Discussion

In the current studies Y2H screening, co-immunoprecipitation, double immunofluorescence labeling and functional studies were performed to identify pendrin binding proteins in the kidney. Our studies demonstrated that IQGAP1, a known scaffolding protein, binds to pendrin in the kidney with strong affinity (Figure 1). Immunofluorescence labeling studies demonstrated co-localization of pendrin and IQGAP1 on the apical membrane of B-intercalated cells (Figure 2A). In addition to the apical membrane, IQGAP1 also shows basolateral distribution pattern in B-intercalated cells (Figure 2A). Co-immunoprecipitation studies revealed that pendrin binds and co-precipitates with IQGAP1 (Figure 3). IQGAP1 shows a strong basolateral expression in a majority of cells in the CCD. The co-localization studies with H^+^-ATPase and IQGAP1 antibodies suggest that these cells are predominantly A-intercalated cells (Figure 2B).

Previous immunohistochemical staining studies localized IQGAP1 to the basolateral membrane of cells in the CCD and several other nephron segments (Lai, et al., 2008). Our studies clearly confirm this finding with IQGAP1 exhibiting abundant expression in the CCD (Figure 2). There were 2 distinct patterns of expression for IQGAP1 in the collecting duct. IQGAP1 is predominantly localized to the basolateral membrane of intercalated cells, and also on the apical membrane of B-intercalated cells (Figure 2). The localization of IQGAP1 in principal cells remains conflicting, with previous studies showing a cytoplasmic pattern and our studies indicating a mixture of cytoplasmic and basolateral labeling (personal observation on AQP-2 and IQGAP1 double-labeling). The specific expression pattern of IQGAP1 in CCD cells suggest that this scaffolding protein, through its differential localization in specific cell populations, may play important roles in determining the cell specific localization of transporters and tubular functions.

Published reports indicate that as a scaffolding protein, IQGAP1 is important in cell differentiation, proliferation, cell polarity and cell-cell adhesion (White et al., 2012; Hedman et al., 2015). Through its IQ motifs, IQGAP1 binds to epidermal growth factor receptor (EGFR), which is responsible for maintaining IQGAP1 in the basolateral membrane domain (White, et al., 2012; Nammalwar, et al., 2015; Nauert, et al., 2003; Jacquemet, et al., 2013; Johnson, et al., 2013; Hedman, et al., 2015). EGFR is known to play an important role in salt and water reabsorption through the epithelial sodium channel (ENaC) and AQP-2 in principal cells, where IQGAP1 is detected on their basolateral membrane domain (Kwakkenbos, et al., 2004; Cheung, et al., 2016). How much of these EGFR regulatory functions require its interaction with IQGAP1 remains speculative. Further, the role of apical IQGAP1 and its binding with pendrin in bicarbonate secretion and chloride absorption in B-intercalated cells requires further investigation.

The cytoplasmic C-terminus of pendrin and the other nine members of SLC26 members is largely comprised of a STAS domain (Bonar and Casey, 2008; Sindić, et al., 2007; Soleimani, 2013; Alper and Sharma, 2013). Mutations in some of the SLC26 genes cause hereditary recessive disorders, including chondrodysplasia (SLC26A2/DTD), chloride-losing diarrhea (SLC26A3/DRA), and Pendred Syndrome (SLC26A4/pendrin) (Bonar and Casey, 2008; Sindić, et al., 2007; Soleimani, 2013; Alper and Sharma, 2013). Many of these mutations involve the respective STAS domains. The C-terminus fragment used for our Y2H expression studies encompasses a number of mutations that are associated with Pendred syndrome and EVA/DFNB4 (Dossena, et al., 2011; Roesch, et al., 2021). This fragment includes the entire STAS domain, strongly suggesting that IQGAP1 is binding to the pendrin STAS domain.

Functional studies in cultured cells indicated that co-expression of IQGAP1 with SLC26A4 enhanced Cl^−^/HCO_3_^−^ exchanger activity mediated *via* SLC26A4, and confocal microscopy showed increased membrane expression of pendrin in the presence of IQGAP1, consistent with enhanced trafficking to the membrane (Figure 4D). While the membrane localization of pendrin is enhanced by co-expression of IQGAP1 and may lead to increased transport function, the possibility that other signaling pathways may contribute to enhanced pendrin activity could not be excluded. Our studies suggest that the interaction of pendrin and IQGAP1 may play an important role in the cell surface localization of pendrin (Figure 4D). Previous studies have shown that IQGAP1 is associated with the actin cytoskeleton and enhances the cross-linking of actin (Bashour et al., 1997; Fukata, et al., 1997). IQGAP1 interaction and crosslinking of the actin cytoskeleton depends on its multimerization mediated *via* binding with the RHO-GTPase family proteins, RAC1 and CDC2 (Fukata et al., 1997). IQGAP1 interacts with other proteins such as ezrin, a membrane F-actin linker protein, and nephrin, a component of slit diaphragm of podocytes, both of which bind to the actin cytoskeleton in polarized cells and may act as nucleation hubs for the formation of signaling complexes (Liu et al., 2015; Nammalwar et al., 2015). Studies by Russo et al. (2017) also demonstrated the RhoA dependent interaction of pendrin with F-actin in cultured bronchiolar cells. The above studies indicate that both pendrin and IQGAP1 interact with the actin cytoskeleton. The latter observations, as well as the direct binding of pendrin and IQGAP1 (documented in this manuscript), support a view that IQGAP1 may play an important role in the regulation of localization and function of SLC26A4.

In conclusion, IQGAP1 co-localizes with SLC26A4 (pendrin) on the apical membrane of B-intercalated cells. IQGAP1 enhances the membrane expression and activity of SLC26A4 in cultured cells. We propose that SLC26A4 interaction with IQGAP1 could play an important role in the regulation of CCD function and physiology, and that disruption of this interaction may contribute to altered SLC26A4 trafficking and/or activity in pathophysiologic states.

## Data Availability

The original contributions presented in the study are included in the article/Supplementary Material, further inquiries can be directed to the corresponding author.
