# Supplementary figures and images for: Identification of IQGAP1 as a SLC26A4 (Pendrin)-Binding Protein in the Kidney
Source: Front Mol Biosci. 2022 May 5;9:874186. doi: 10.3389/fmolb.2022.874186 (PMC9117723; doi:10.3389/fmolb.2022.874186)

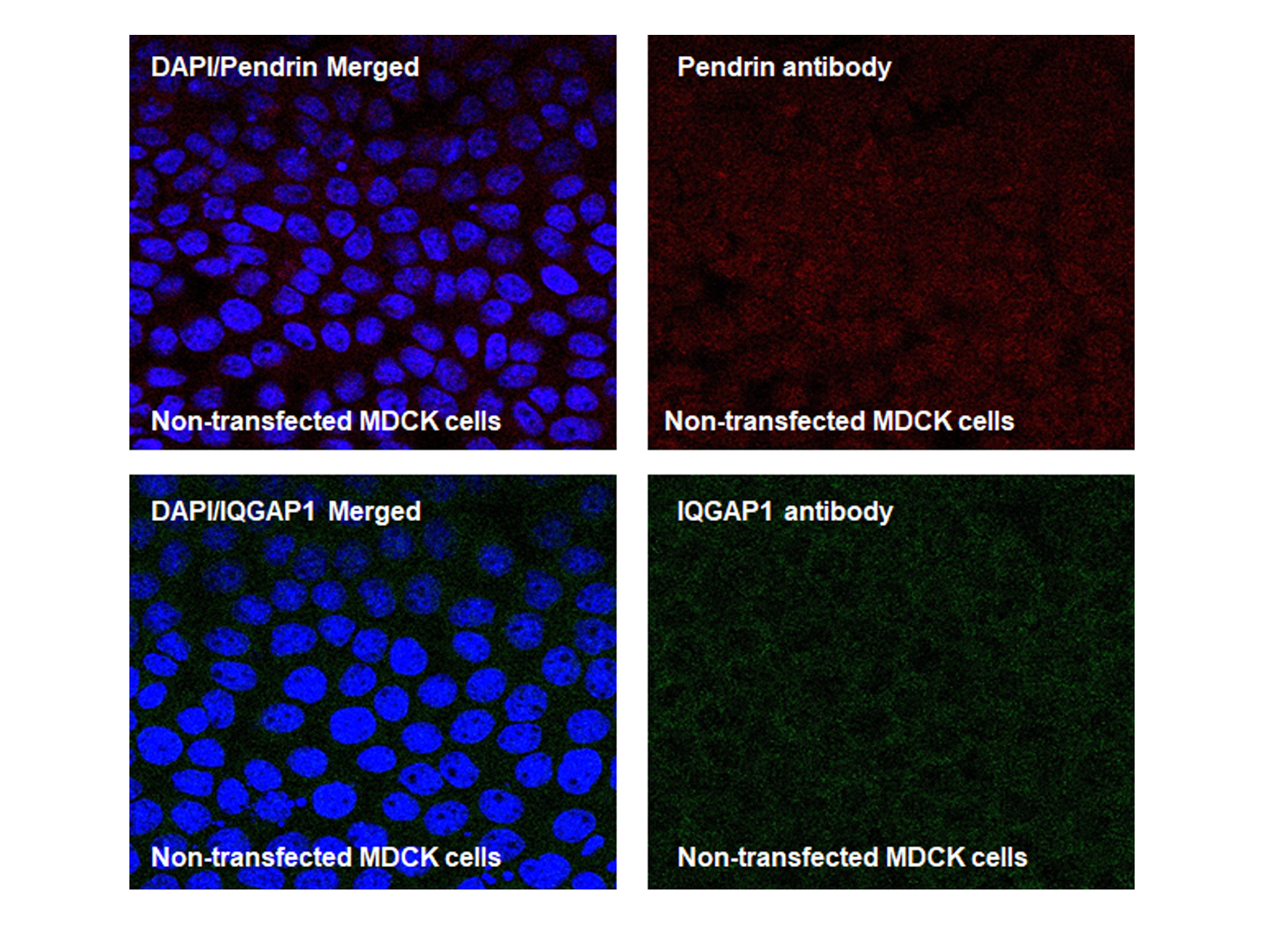

Supplement: Supplementary file 1 [file Image1.jpg]
